# A Fluorescent Linear Conjugated Polymer Constructed from Pillararene and Anthracene

**DOI:** 10.3390/molecules27103162

**Published:** 2022-05-15

**Authors:** Dinghui Wang, Jun Wang, Yan Wang, Yingwei Yang

**Affiliations:** International Joint Research Laboratory of Nano-Micro Architecture Chemistry, College of Chemistry, Jilin University, 2699 Qianjin Street, Changchun 130012, China; wangdh19@mails.jlu.edu.cn (D.W.); junw19@mails.jlu.edu.cn (J.W.)

**Keywords:** conjugated polymer, host–guest interaction, pillararene, supramolecular chemistry, light-emitting materials

## Abstract

Over the past few years, conjugated polymers (CPs) have aroused much attention owing to their rigid conjugated structures, which can perform well in light harvesting and energy transfer and offer great potential in materials chemistry. In this article, we fabricate a new luminescent linear CP p(P[5](OTf)_2_-*co*-9,10-dea) via the Sonogashira coupling of 9,10-diethynylanthracene and trifluoromethanesulfonic anhydride (OTf) modified pillar[5]arene, generating enhanced yellow-green fluorescence emission at around 552 nm. The reaction condition was screened to get a deeper understanding of this polymerization approach, resulting in an excellent yield as high as 92% ultimately. Besides the optical properties, self-assembly behaviors of the CP in low/high concentrations were studied, where interesting adjustable morphologies from tube to sheet were observed. In addition, the fluorescence performance and structural architecture can be disturbed by the host–guest reorganization between the host CP and the guest adiponitrile, suggesting great potential of this CP material in the field of sensing and detection.

## 1. Introduction

Compared with traditional flexible non-conjugated polymers and small conjugated molecules, conjugated polymers (CPs) containing rigid conjugated backbones have outstanding capability of light harvesting and energy transfer, favorable to the generation of luminescence, thus endowing widespread applications in electronic sensing devices, solar battery, and semiconductor materials [1,2,3,4,5,6,7,8,9,10]. Consequently, it is essential to work out facile and powerful strategies for obtaining promoted light-emitting CPs with high efficiency [11,12,13,14,15,16,17].

Supramolecular assemblies have been widely reported to assist in constructing CP-based artificial light-harvesting systems [18]. With the rapid development of supramolecular chemistry, various macrocyclic compounds with rigid structures have been designed and synthesized [19,20,21,22,23]. Benefiting from their facile synthesis, high yield, rigid cavity structures, and easy modification, pillararenes and their derivatives, as a relatively new class of synthetic macrocycles, have been widely applied for the design of high-performance fluorescent supramolecular polymeric materials [24,25,26,27,28], guaranteeing a variety of applications in fluorescence imaging [29,30], substance detection [31,32], electroluminescence materials [33,34,35,36,37,38], and fluorescence sensor [39,40,41,42]. For example, our group reported one conjugated macrocycle polymer (CMP) from tetraphenylethene (TPE) modified pillar[5]arene, which could be manufactured into a well-behaved fluorescence sensor to detect Fe^3+^ and 4-aminoazobenzene, suggesting promising potential for the removal of environmental pollutants [43]. In addition, the applications of CMP materials in catalysis, sensing, adsorption, and separation have been studied in detail [44,45,46,47,48,49,50,51]. However, common CMP materials show limitations in solubility due to the strongly cross-linked structures, which leads to weak processability. To overcome this drawback, linear CPs containing pillararenes have been obtained through Suzuki coupling by Tang and coworkers [52]. Instead of covalently bonding with pillararenes, the guest TPE molecules were brought into the linear backbone successfully through the encapsulation of the host pillararenes. This system not only showed a highly efficient light-harvesting property with fluorescence enhancement but also promised excellent solubility in CHCl_3_/c-hexane. Our research group also reported two kinds of linear CPs constructed from terphenyl and diphenyl-pillararenes and their applications in photocatalysis on the basis of the enlarged visible-light absorption region and tunable optoelectronic properties [1].

To enrich the synthetic methods and stimulate the property improvement of CPs, several methods have been utilized to construct linear CPs, including Suzuki coupling [53], Heck reaction [54], Negishi coupling [55], Stille coupling [56], and Sonogashira coupling [57]. Benefiting from the co-catalysis of copper and palladium, Sonogashira coupling as one of the essential methods for the palladium-catalyzed coupling reaction of halogenated aryl compounds with terminal alkynes shows the advantages of high yields and mild reaction conditions, enabling the synthesis of linear CPs with good solubility and high machinability [58].

**Scheme 1:**
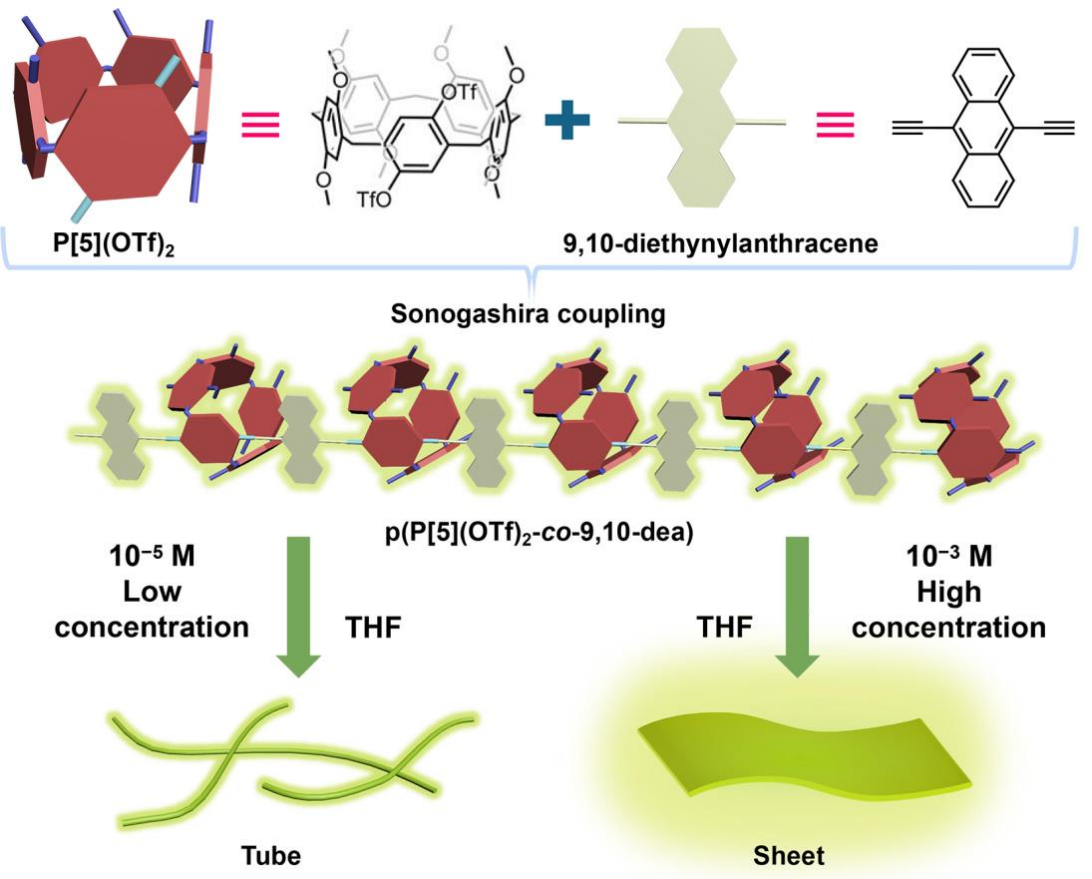
Schematic representation of the linear fluorescent CP, p(P[5](OTf)_2_-co-9,10-dea), and its tunable morphology under different concentrations.

Herein, we fabricate a new linear fluorescent CP, p(P[5](OTf)_2_-*co*-9,10-dea), from the Sonogashira coupling of trifluoromethanesulfonic anhydride (OTf)-modified pillar[5]arene and 9,10-diethynylanthracene containing alkynyl, which could generate strengthened yellow-green fluorescence emission at around 552 nm in THF (Scheme 1). Notably, the best reaction conditions of Sonogashira coupling were selected by adopting the control variable method. Moreover, tunable fluorescence intensity and morphologies could be realized by varying the solution concentration of p(P[5](OTf)_2_-*co*-9,10-dea) or the guest adiponitrile.

## 2. Results and Discussion

### 2.1. Polymerization

According to the previously published procedures, two reactants, that is P[5](OTf)_2_ and 9,10-diethynylanthracene, and the CP (p(P[5](OTf)_2_-*co*-9,10-dea) were synthesized and characterized by ^1^H nuclear magnetic resonance (^1^H NMR) and ^13^C NMR (Scheme S1 and Figures S1–S8) [59,60]. Then the key parameters of Sonogashira coupling, involving solvent, reaction time, temperature, and the feed ratio of catalyst, were profoundly studied to achieve the highest polymerization yield of this new system; the detailed contents are as follows.

Firstly, four kinds of organic solvents were selected according to the reported Sonogashira coupling systems to investigate the solvent effect [61]. As shown in Table 1, the solvents all ensured the polymerization in good progress, among which THF was identified as the most suitable one for the polymerization, allowing the highest yield up to 92% (Table 1, Entry 2).

Then, the influence of reaction time was systematically discussed in the most optimized solvent THF (Table 2). The polymeric product started to yield after 8 h with a conversion rate of 27% (Table 2, Entry 1). Prolonging the reaction time to 24 h kept raising the yield to 92% (Table 2, Entries 2–5). However, further increased duration of polymerization to more than 24 h was not favorable for the formation of the CP because the decreased activity of the catalyst caused by the complex of CuI and 9,10-diethynylanthracene resulted in reduced yields (Table 2, Entries 6 and 7) [62]. Therefore, 24 h was preferred as the optimum reaction time in subsequent studies.

Afterward, the temperature effect on the polymerization was investigated. Due to the bad reactivity of substituted aromatic hydrocarbons at low temperatures (≤60 °C), it is necessary to elevate the chemical reactivity by increasing the reaction temperature (Table 3, Entries 1 and 2). Upon raising the temperature to 70 °C, the CP started to generate with a yield of 18% (Table 3, Entry 3). Upon increasing the temperature to 90 °C, further production increment was achieved (92%, Table 3, Entries 4–7). However, when conducting the reaction at 100 °C, a slightly reduced yield was observed (Table 3, Entry 8). We rationally inferred that this negative result ascribes to the unnecessary side reactions in high-temperature environments [63]. Since too high and too low temperatures are not favorable for the Sonogashira coupling, we chose 90 °C as the ideal reaction temperature.

Last but not least, the amount of CuI as the co-catalyst is vital to the reduction reaction of the palladium catalyst in the Sonogashira coupling. Nevertheless, excessive CuI results in the complex formation with 9,10-diethynylanthracene, minimizing reactant concentration. As we assumed, the polymer p(P[5](OTf)_2_-*co*-9,10-dea) engendered only in 0.03 M (79%), 0.04 M (92%), and 0.05 M (71%) (Table 4), where 0.04 M CuI presents the best co-catalyst concentration for the following polymerization.

### 2.2. Structural Characterization

**Figure 1:**
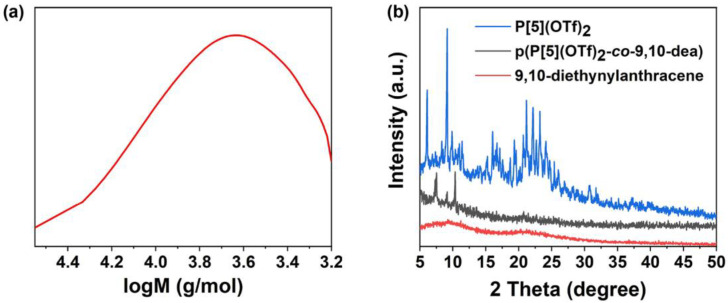
(**a**) GPC trace of p(P[5](OTf)_2_-*co*-9,10-dea). (**b**) XRD patterns of P[5](OTf)_2_ (blue), p(P[5](OTf)_2_-*co*-9,10-dea) (black), and 9,10-diethynylanthracene (red).

Compared with the previously reported CMP materials and heteroatoms-containing CPs, the new linear p(P[5](OTf)_2_-*co*-9,10-dea) obtained under the optimized reaction condition combines the superiorities of conjugate rigid plane structure and good solubility, which enables its characterization in solution. Firstly, we characterized the number of average molecular weight (Mn) and the polymer dispersity index (PDI) by gel permeation chromatography (GPC). As illustrated in Figure 1a and Figure S9, Mn and PDI were calculated to be 4103 and 1.46, respectively, revealing 4–5 repeating units in one single chain of the resulting CP. This is in good agreement with the literature reports that the strengthened steric hindrance effect of reactants always results in short chains [64,65,66,67]. The Mn calculated from the ^1^H NMR spectrum of p(P[5](OTf)_2_-*co*-9,10-dea) is 3798, which coincides well with the GPC result. Then, X-ray diffraction (XRD) pattern showed the disappearance of P[5](OTf)_2_ peaks. New peaks emerged in p(P[5](OTf)_2_-*co*-9,10-dea), indicating its crystalline structure (Figure 1b).

**Figure 2:**
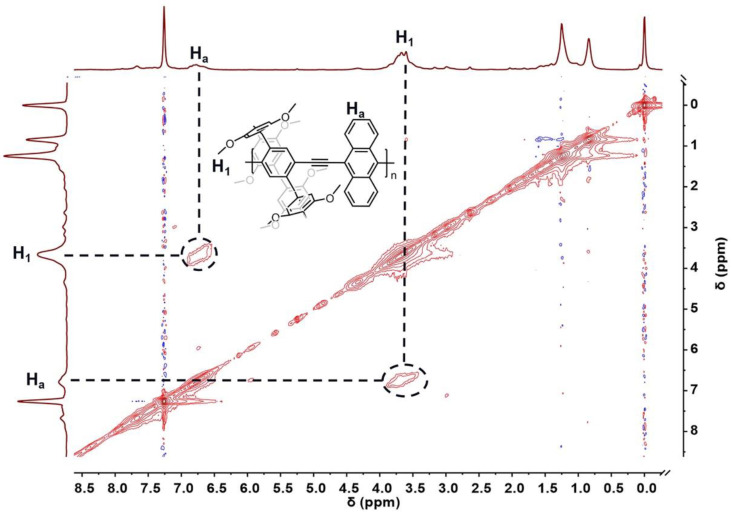
Two-dimensional ROESY spectrum (600 MHz, CDCl_3_, 298 K) of p(P[5](OTf)_2_-*co*-9,10-dea).

The 2D rotating frame Overhauser effect spectroscopy nuclear magnetic resonance (2D ROESY NMR) experiment of p(P[5](OTf)_2_-*co*-9,10-dea) supports the correlations between the methoxy moieties at around 3.44–3.93 ppm (H_1_) and the phenyl protons at around 6.50–6.93 ppm (H_a_), demonstrating the strong intermolecular interactions between the polymer chains (Figure 2).

### 2.3. Fluorescence Properties

**Figure 3:**
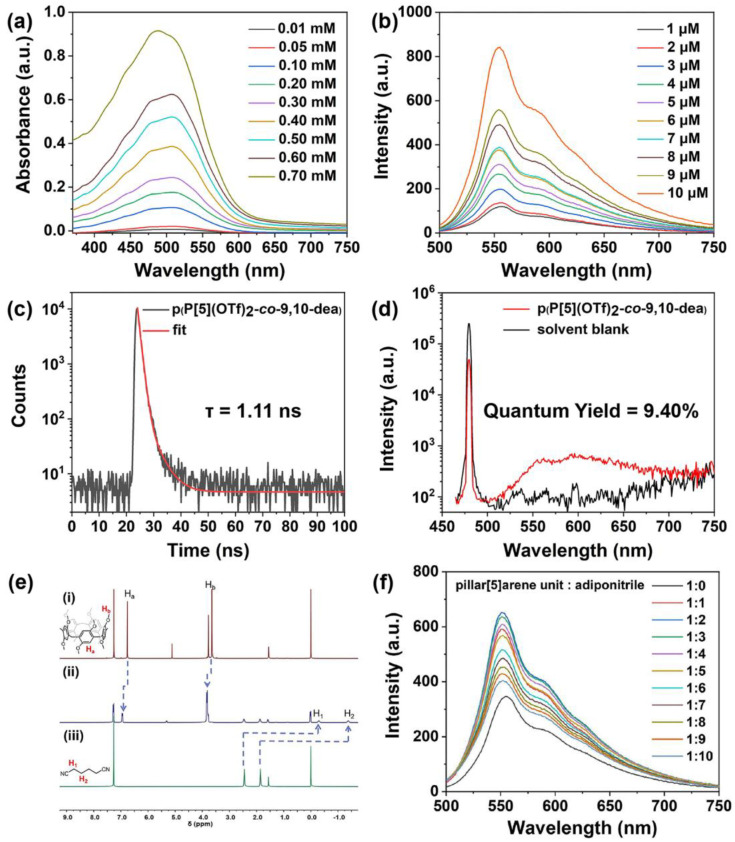
(**a**) The UV-vis absorption spectra and (**b**) fluorescence spectra of p(P[5](OTf)_2_-co-9,10-dea) CP in THF with increasing concentration (slit widths: ex. 5 nm, em. 3 nm; 25 °C). (**c**) Fluorescence decay profile and (**d**) quantum yield of p(P[5](OTf)_2_-co-9.10-dea) (solvent: THF, λ_ex_ = 480 nm; [pillar[5]arene unit] = 10–5 M, 25 °C). (**e**) Characterization of the host–guest interactions between pillar[5]arenes and adiponitrile guests by NMR spectroscopy. ^1^H NMR spectra (400 MHz, CDCl_3_, 298 K) of (i) DMP[5]A (5 × 10^−3^ M), (ii) the equimolar mixture of DMP[5]A (5 × 10^−3^ M) and adiponitrile (5 × 10^−3^ M), (iii) adiponitrile (5 × 10^−3^ M). (**f**) Fluorescent emission spectra of p(P[5](OTf)2-co-9.10-dea) in THF with different ratio of adiponitrile (λ_ex_ = 480 nm; λ_em_ = 552 nm; slit widths: ex. 5 nm, em. 3 nm; 25 °C).

As the reaction proceeded, the conjugated structure was further extended, which increased electron delocalization and reduced the energy required for electron transitions, thus resulting in the redshift of the ultraviolet-visible (UV-vis) absorption band and the corresponding fluorescence emission band to 480 nm and 552 nm, respectively (Figure 3a,b and Figures S10–S12). When increasing the CP concentration in THF, the UV-vis absorption and fluorescence intensities were all raised. Nevertheless, after introducing the poor solvent H_2_O to the solution, fluorescence intensity decreased significantly, revealing quenching from π–π stacking of the polymeric dye (Figure S13) [68]. Additionally, a reduced lifetime of about 1.11 ns and promoted quantum yield of around 9.40% of p(P[5](OTf)_2_-*co*-9,10-dea) solution were produced, which is conducive to the construction of efficient short-frequency emission fluorescent materials (Figure 3c,d, and Figures S14 and S15).

The current pillar[5]arenes with an intrinsic π-electron-rich cavity in the CP chain endow the selective recognition to the cyano group based on the host–guest interaction (Figure 3e). The introduction of suitable guest molecules undoubtedly affects the fluorescence emission of CP. The fluorescence spectra with different ratios of adiponitrile containing cyano groups at chain ends were collected (Figure 3f and Figure S16), which revealed an appreciable nonlinear relationship between the fluorescence intensity and the guest-host ratio within the range from 2:1 to 20:1, offering new possibilities for the application of sensing and detection.

### 2.4. Tunable Morphologies Induced by Concentration

**Figure 4:**
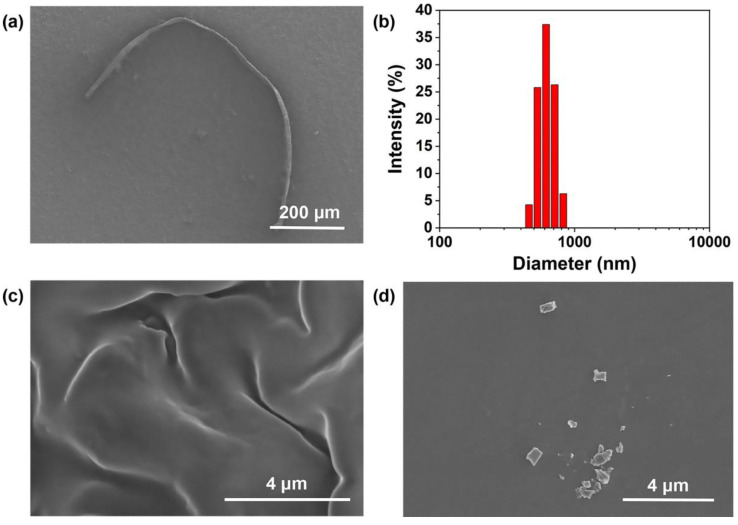
(**a**) SEM image and (**b**) hydrodynamic size of p(P[5](OTf)2-*co*-9,10-dea) in THF, [pillar[5]arene unit] = 10−5 M. (**c**) SEM image of p(P[5](OTf)2-*co*-9,10-dea) in THF ([pillar[5]arene unit] = 10−3 M) and (**d**) adiponitrile and p(P[5](OTf)2-*co*-9,10-dea) in THF ([pillar[5]arene unit]: [adiponitrile] = 1:10).

To comprehensively understand this new CP, we continue to investigate its assembly structures at different concentrations by the scanning electron microscope (SEM) and dynamic light scattering (DLS) experiments. At a low concentration of 1 × 10^−5^ M, p(P[5](OTf)_2_-*co*-9,10-dea) assembled into a linear long-chain structure of about 970 μm (Figure 4a). The narrow distributed hydrodynamic radius was found to be 615 nm, demonstrating the good dispersibility and stability of this CP in THF (Figure 4b). As shown in Figure 4c, when increased the concentration to 1 × 10^−3^ M, p(P[5](OTf)_2_-*co*-9,10-dea) changed its self-assembled structure to a wrinkled film due to the amplified intermolecular interactions as we characterized above. This interesting transformation provides an idea for building fluorescent systems with controllable structures. In addition, the structures were disturbed after the combination of p(P[5](OTf)_2_-*co*-9,10-dea) and adiponitrile. The formation of the supramolecular polymer limited the aggregation of the CP with planar structure, forming into irregular blocks (Figure 4d).

## 3. Materials and Methods

### 3.1. Materials

All the reagents and solvents were purchased from commercial sources and used as received unless otherwise noted. Deionized water, purified by Experimental Water System (Lab-UV-20), was used in the relevant experiments.

### 3.2. Physical Characterization and Techniques

^1^H NMR and ^13^C NMR spectra were recorded on a Bruker Avance 400 MHz spectrometer, and 2D ROESY NMR spectra were recorded on a Bruker AVANCEIII 600 MHz instrument. UV-vis spectra were recorded on a Shimadzu UV-2550 instrument. XRD measurements were carried out on a PANalytical B.V. Empyrean powder diffractometer. GPC was performed on a Malvern instrument, equipped with a PLgel MIXED guard column followed by a PLgel MIXED guard column (molecular weight range 2.0 × 10^2^–4.0 × 10^5^ g/mol), column thermostated to 60 °C, and calibrated by linear polystyrene standards. THF was used as the eluent at a flow of 0.8 mL/min at 60 °C. The fluorescent experiments were conducted on an RF-5301 spectrofluorophotometer (Shimadzu Corporation, Kyoto, Japan). The time-resolved fluorescence decay curves and fluorescence quantum yields were obtained on an FLS920 instrument (Edinburgh Instrument, Livingston, UK). DLS was obtained on a Zetasizer Nano ZS instrument. Scanning electron microscope (SEM) images were obtained on a HITACHI-SU8082 instrument.

## 4. Conclusions

In this work, we successfully designed and prepared a new type of CP with linear structure and good solubility via the Sonogashira coupling, achieving a polymerization yield as high as 92% after optimizing the reaction parameters. Notably, promoted red-shifted yellow-green fluorescence at around 552 nm was harvested in THF. When regulating the concentration/adding the guest adiponitrile, diverse fluorescence intensity and self-assembly structures from line to film/irregular blocks were attained when regulating the concentration/adding the guest adiponitrile. In general, this fluorescent linear CP with concentration/adiponitrile-dependent luminescence properties and self-assembly characteristics is appropriate for sensing and available in the fabrication of well-performing light harvesting and energy transfer pure organic materials with controllable structures.

## Figures and Tables

**Table 1:** Solvent effect on the polymerization carried out under N_2_ atmosphere for 24 h in the presence of CuI and Et_3_N. T = 90 °C; [P[5](OTf)_2_] = [9,10-diethynylanthracene] = 0.30 M; [Pd(PPh_3_)_2_Cl_2_] = 0.02 M; [PPh_3_] = 0.03 M; [CuI] = 0.04 M; Et_3_N = 0.6 mL.

Entry	Solvent	Yield (%)
1	CHCl_3_	88
2	THF	92
3	DMF	72
4	CH_2_Cl_2_	74

**Table 2:** Time course of the polymerization carried out under N_2_ atmosphere in the presence of CuI and Et_3_N in THF. T = 90 °C; [P[5](OTf)_2_] = [9,10-diethynylanthracene] = 0.30 M; [Pd(PPh_3_)_2_Cl_2_] = 0.02 M; [PPh_3_] = 0.03 M; [CuI] = 0.04 M; Et_3_N = 0.6 mL.

Entry	Reaction Time (h)	Yield (%)
1	8	27
2	10	43
3	11	60
4	12	77
5	24	92
6	36	91
7	48	82

**Table 3:** Temperature effect on the polymerization carried out under N_2_ atmosphere for 24 h in the presence of CuI and Et_3_N in THF. [P[5](OTf)_2_] = [9,10-diethynylanthracene] = 0.30 M; [Pd(PPh_3_)_2_Cl_2_] = 0.02 M; [PPh_3_] = 0.03 M; [CuI] = 0.04 M; Et_3_N = 0.6 mL.

Entry	Reaction Temperature (°C)	Yield (%)
1	25	/
2	60	/
3	70	18
4	75	34
5	80	59
6	85	79
7	90	92
8	100	89

**Table 4:** Effect of catalyst loading on the polymerization carried out under N_2_ atmosphere for 24 h in the presence of CuI and Et_3_N in THF. T = 90 °C; [P[5](OTf)_2_] = [9,10-diethynylanthracene] = 0.30 M; [Pd(PPh_3_)_2_Cl_2_] = 0.02 M; [PPh_3_] = 0.03 M; Et_3_N = 0.6 mL.

Entry	CuI (M)	Yield (%)
1	0.10	/
2	0.05	79
3	0.04	92
4	0.03	71
5	0.02	/
6	0.01	/

## Data Availability

Not applicable.

## Supplementary Materials

The following supporting information can be downloaded at: https://www.mdpi.com/article/10.3390/molecules27103162/s1, Figures S1–S9: Synthetic routes and structural characterization of compounds and polymer; Figures S10–S17: The luminescence spectra of materials.

## Sample Availability

Samples of the compounds are not available from the authors.
