# Supplementary material for: A Fluorescent Linear Conjugated Polymer Constructed from Pillararene and Anthracene
Source: Molecules. 2022 May 15;27(10):3162. doi: 10.3390/molecules27103162 (PMC9146593; doi:10.3390/molecules27103162)
Supplement: Supplementary file 1 [file molecules-27-03162-s001.zip › molecules-1714340-supplementary.pdf]

Supplementary Materials

A Fluorescent Linear Conjugated Polymer Constructed from Pillararene and Anthracene

Dinghui Wang, Jun Wang, Yan Wang*, Yingwei Yang*

International Joint Research Laboratory of Nano-Micro Architecture Chemistry, College of Chemistry, Jilin University, 2699 Qianjin Street, Changchun, 130012, PR China; wangdh19@mails.jlu.edu.cn; junw19@mails.jlu.edu.cn

* Correspondence: wangy2011@jlu.edu.cn; ywyang@jlu.edu.cn

Synthetic protocols of p(P[5](OTf)2-*co*-9,10-dea)

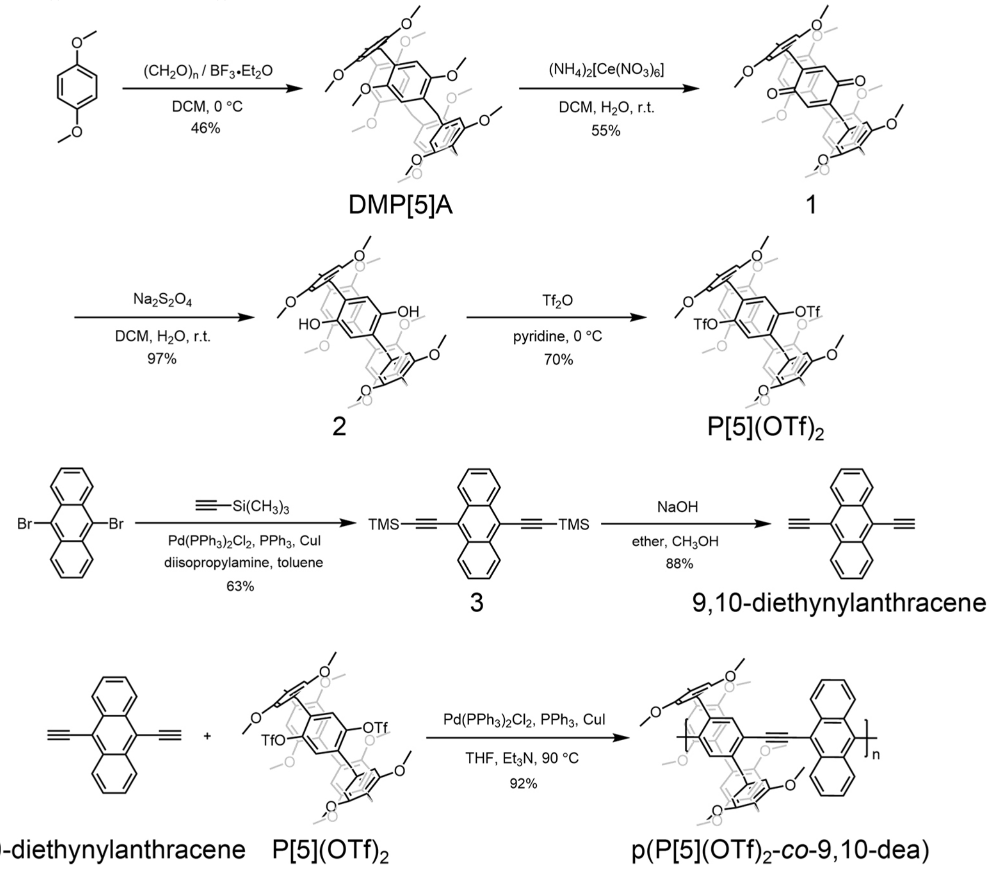

Scheme S1. Synthetic route to p(P[5](OTf)2-*co*-9,10-dea).

Synthesis of DMP[5]A

1,4-Dimethoxybenzene (14 g, 50 mmol), $(CH_2O)_n$ (9 g, 150 mmol), and dichloromethane (DCM, 600 mL) were added to a flask with stirring. $BF_3{\cdot}Et_2O$ (14 mL, 132 mmol) was added after 30 min. After the solution color changed from white to light yellow, and then to olivine, finally to dark green (30 min), the reaction was quenched by $NaHCO_3$ aqueous solution and extracted with DCM 3 times. The organic layers were combined and dried with anhydrous

Na_2SO_4 overnight. The solid Na_2SO_4 was removed by filtration. After evaporating the solvent through the rotation-evaporation method, the crude DMP[5]A was obtained, followed by further purification by silica gel column chromatography (PE/DCM, 1:2 v/v as the eluent) to give the product as a white powder (7 g, 46 %). ^{1}H NMR (400 MHz, $CDCl_3$, 298 K), δ (ppm): 6.766 (s, 10H), 3.772 (s, 10H), 3.651 (d, 30H).

Synthesis of compound 1

DMP[5]A (3 g, 4 mmol) was dissolved in DCM (200 mL) under stirring at room temperature. Then $(NH_4)_2[Ce(NO_3)_6]$ (4.38 g, 8 mmol) was added dropwise. The reaction was stopped 40 min later. The mixture was extracted with DCM and washed with NaCl aqueous solution 3 times. The organic extracts were combined and dried over anhydrous $MgSO_4$. After filtering off the solid salt, the crude product was concentrated under a vacuum and further purified by silica gel column chromatography (PE/DCM, 1: 1 v/v as the eluent) to give compound 1 as a red powder (1.6 g, 55%). ^{1}H NMR (400 MHz, $CDCl_3$, 298 K), δ (ppm): 6.841 (s, 2H), 6.806 (d, 4H), 6.662 (s, 4H), 3.786 (t, 6H), 3.743 (s, 6H), 3.714 (d, 12H), 3.626 (d, 6H), 3.589 (s, 4H).

Synthesis of compound 2

Compound 1 (1.4 g, 2 mmol) was dissolved in DCM (20 mL) in a 50 mL round-bottom flask under an N_2 atmosphere. Then an aqueous solution of $Na_2S_2O_4$ (10 mL, 2 M) was added dropwise under vigorous stirring. The reaction was stopped after 30 min. Then the reaction mixture was extracted with DCM and washed with NaCl aqueous solution 3 times. The organic layer was dried over anhydrous $MgSO_4$. After evaporating the DCM (1.4 g, 97%), a white powder was obtained. ^{1}H NMR (400 MHz, DMSO-d_6, 298 K), δ (ppm): 8.199 (s, 2H), 6.768 (m, 10H), 3.654 (m, 34H).

Synthesis of P[5](OTf)$_2$ [S1]

Trifluoromethanesulfonic anhydride (4 mL) was added to DCM containing compound 2 (1.4 g, 2 mmol) and pyridine (dry, 2 mL) at 0 °C. The reaction was stopped after stirring for 12 hours at room temperature. The resulting mixture was extracted with DCM and then washed with NaCl aqueous solution 3 times. The solid raw product was further purified by silica gel column chromatography (PE/DCM, 1: 1 v/v as the eluent) to give a white powder (1.38 g, 70%). ^{1}H NMR (400 MHz, $CDCl_3$, 298 K), δ (ppm): 7.341 (s, 2H), 6.785 (t, 8H), 3.686 (m, 34H).

Synthesis of compound 3

CuI (0.15 g, 0.79 mmol), $Pd(PPh_3)_2Cl_2$ (0.23 g, 0.33 mmol), PPh_3 (0.1 g, 0.38 mmol), 9,10-dibromoanthracene (2.02 g, 6.01 mmol), and TMSA (1.9 g, 19.34 mmol) were dissolved in a mixture of toluene (20 mL) and diisopropylamine (8 mL) in a 100 mL three-necked flask under N_2 atmosphere. After stirring for 16 h at 85 °C, the resulting mixture was extracted with diethyl ether. The organic layer was collected and washed with saturated $NaHCO_3$ solution, then dried over anhydrous $MgSO_4$. Column chromatography was applied with the hexane as eluent to purify the product (1.31 g, 63%, yellow powder). ^{1}H NMR (400 MHz, $CDCl_3$, 298 K), δ (ppm): 8.581 (d, 4H), 7.614 (d, 4H), 0.422 (s, 18H). ^{13}C NMR (100 MHz, $CDCl_3$, 298 K): δ (ppm) 132.044, 126.996, 125.652, 118.239, 107.970, 101.281, 77.128, 77.015, 76.810, 76.493.

Synthesis of 9,10-diethynylanthracene [S2]

Compound 3 (2.0 g, 5.40 mmol), 300 mL fresh diethyl ether, 200 mL methanol, and 100 mL NaOH (1 M) aqueous solution were charged sequentially into a 1000 mL three-necked flask under an N_2 atmosphere. The reaction mixture was stirred for 4 h at room temperature. The resulting mixture was extracted with diethyl ether. The obtained organic solution was dried over anhydrous $MgSO_4$. After evaporation, the product was recrystallized from hexane. (1.08 g, 88%, yellow crystal). ^1H NMR (400 MHz, $CDCl_3$, 298 K), δ (ppm): 8.621 (d, 4H), 7.631 (d, 4H), 4.070 (s, 2H).

Synthesis of poly(P[5](OTf)$_2$-co-9,10-diethynylanthracene)

Poly(P[5](OTf)$_2$-*co*-9,10-diethynylanthracene) was prepared via the Sonogoshira coupling which was initiated by 9,10-diethynylanthracene. In a typical polymerization process, the feed molar ratio of [9,10-diethynylanthracene]: [P[5](OTf)$_2$]: [Pd(PPh_3)$_2$$Cl_2$]: [CuI]: [$PPh_3$] was maintained at 20:20:1:3:2, P[5](OTf)$_2$ (437.2 mg, 0.4 mmol), Pd(PPh_3)$_2$$Cl_2$ (15.5 mg, 0.02 mmol), CuI (12.64 mg, 0.066 mmol), PPh_3 (11.6 mg, 0.044 mmol), Et_3N (0.6 mL) and THF (0.6 mL) were added to a branch-necked flask, degassing under three freeze-vacuate-thaw cycles. The mixture was heated at 90 °C. The 9,10-diethynylanthracene (100 mg, 0.4 mmol) in THF (0.3 mL) was injected into the flask to initiate the reaction. After the reaction was conducted for 24 h, poly(P[5](OTf)$_2$-*co*-9,10-diethynylanthracene) (p(P[5](OTf)$_2$-*co*-9,10-dea)) was precipitated in MeOH and acetone for 3 times and dried in vacuum. ^1H NMR (400 MHz, $CDCl_3$, 298 K), δ (ppm): 8.799 (d, 10H), 6.781 (s, 8H), 3.682 (m, 34H).

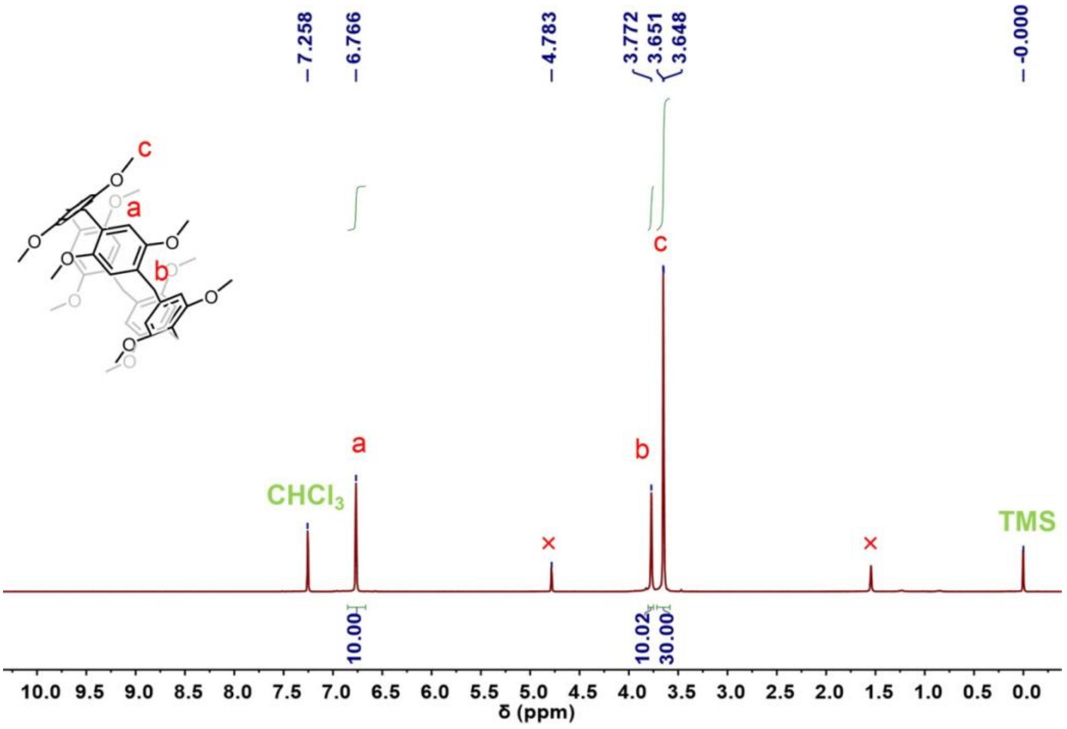

Figure S1. ^1H NMR spectrum (400 MHz, $CDCl_3$, 298 K) of DMP[5]A.

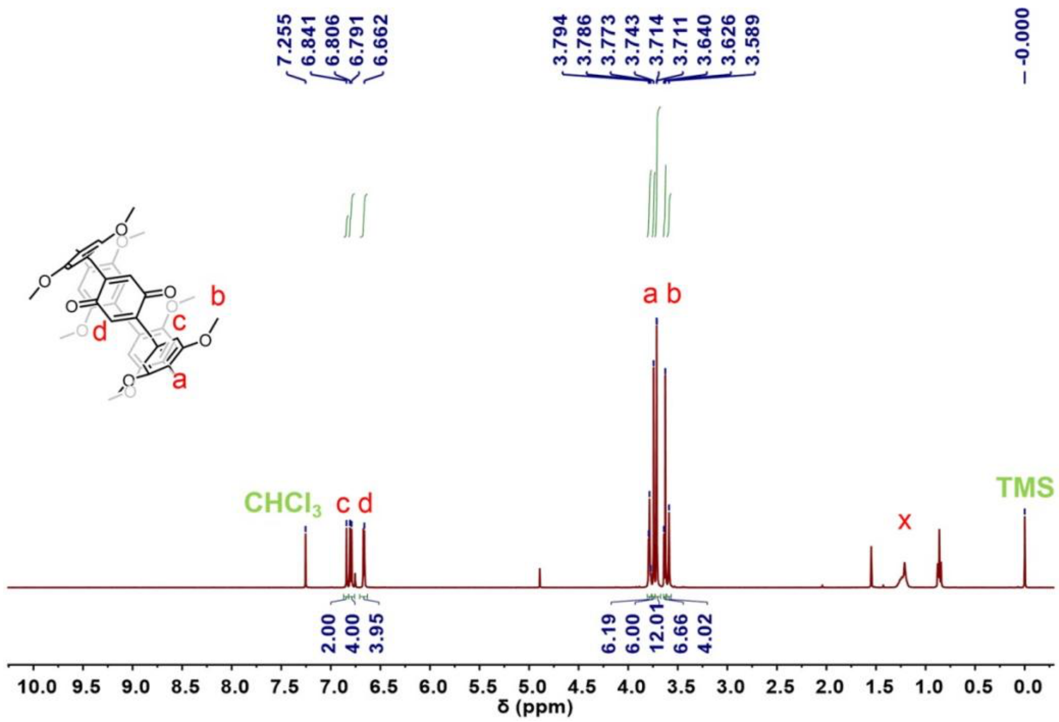

Figure S2. ^{1}H NMR spectrum (400 MHz, $CDCl_3$, 298 K) of compound 1.

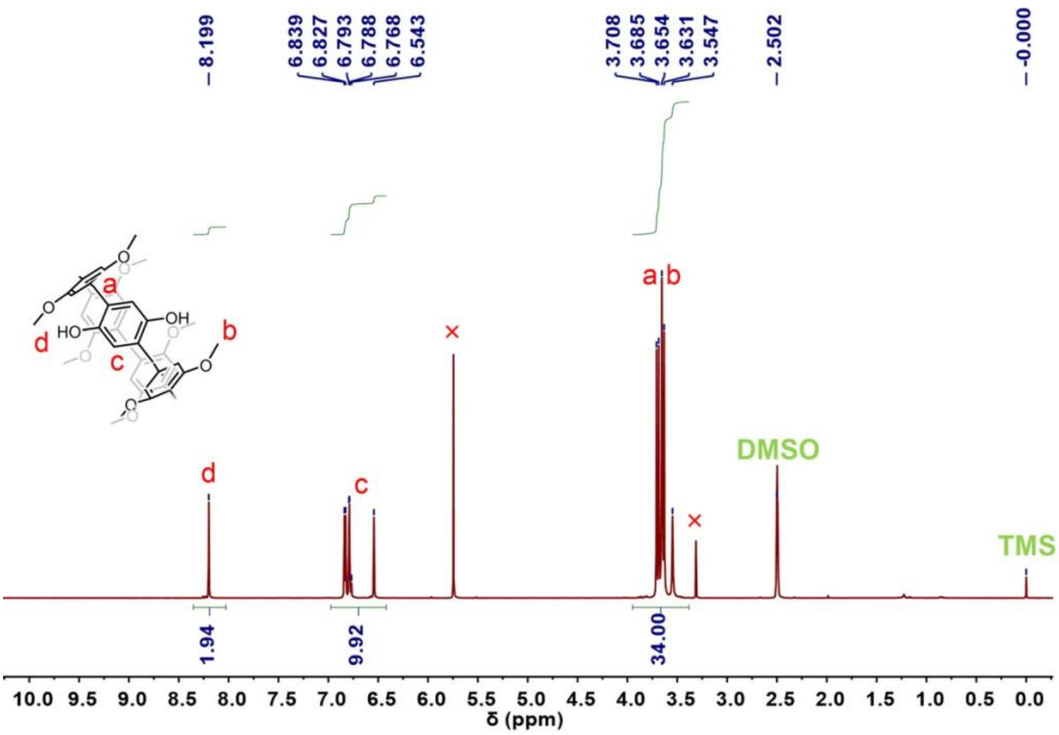

Figure S3. ^{1}H NMR spectrum (400 MHz, DMSO-d_6, 298 K) of compound 2.

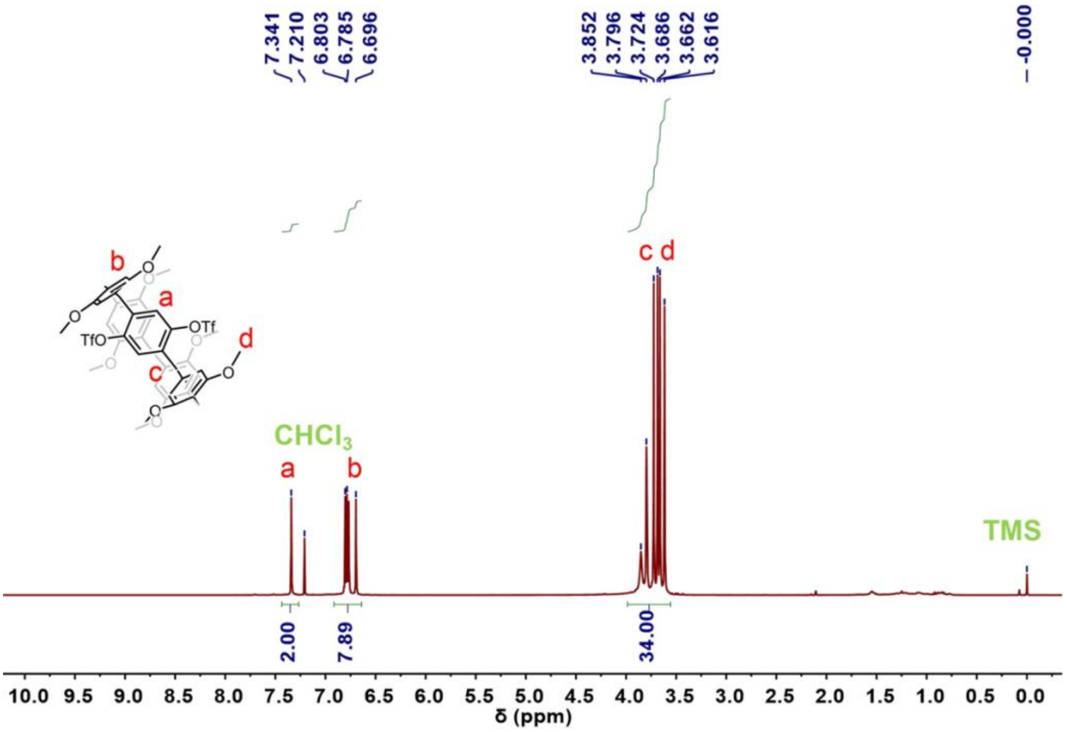

Figure S4. ^1H NMR spectrum (400 MHz, $CDCl_3$, 298 K) of P[5](OTf)$_2$.

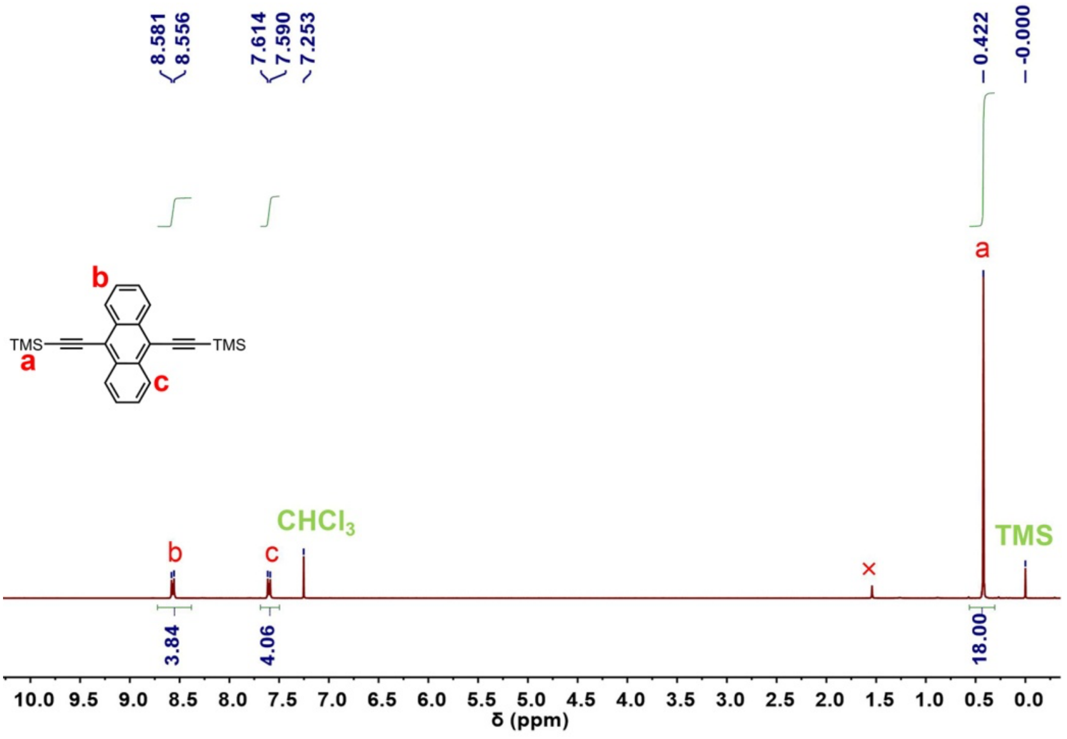

Figure S5. ^1H NMR spectrum (400 MHz, $CDCl_3$, 298 K) of compound 3.

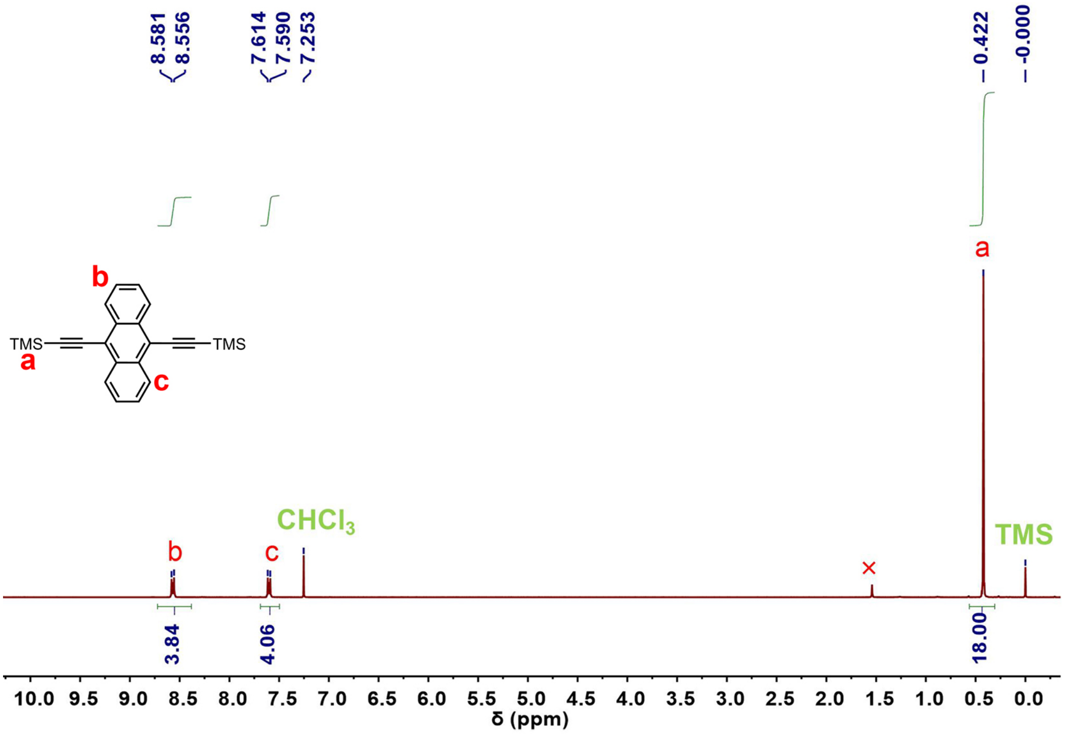

Figure S6. ^{13}C NMR spectrum (100 MHz, $CDCl_3$, 298 K) of compound 3.

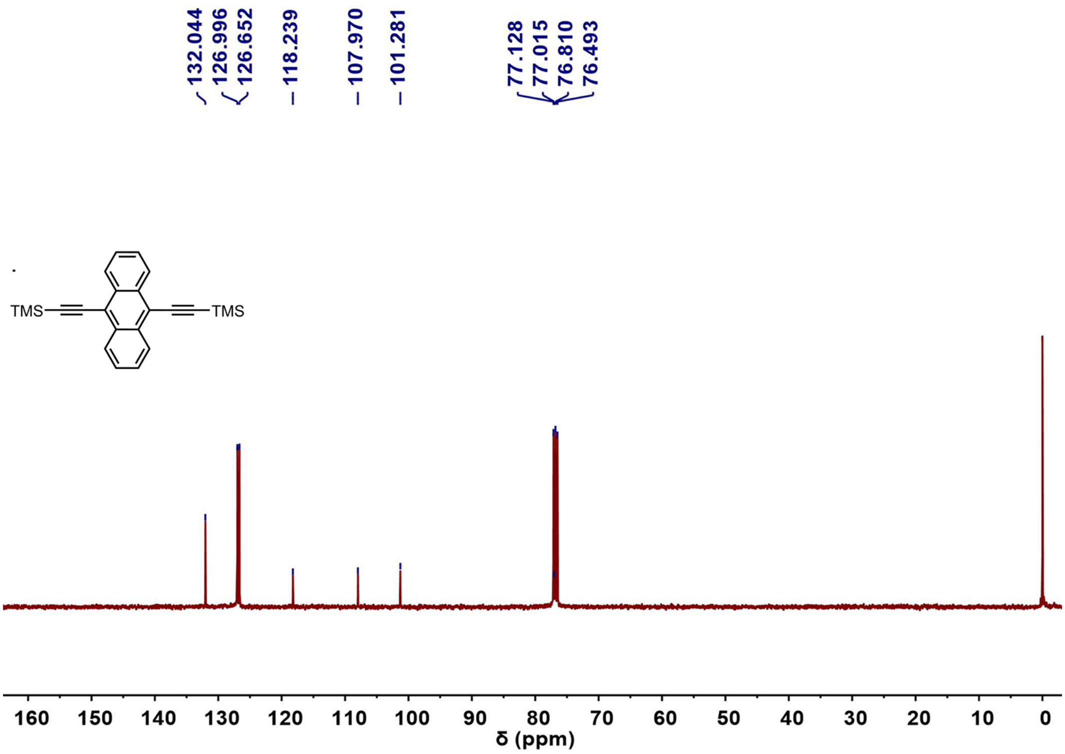

Figure S7. ^{1}H NMR spectrum (400 MHz, $CDCl_3$, 298 K) of 9,10-diethynylanthracene.

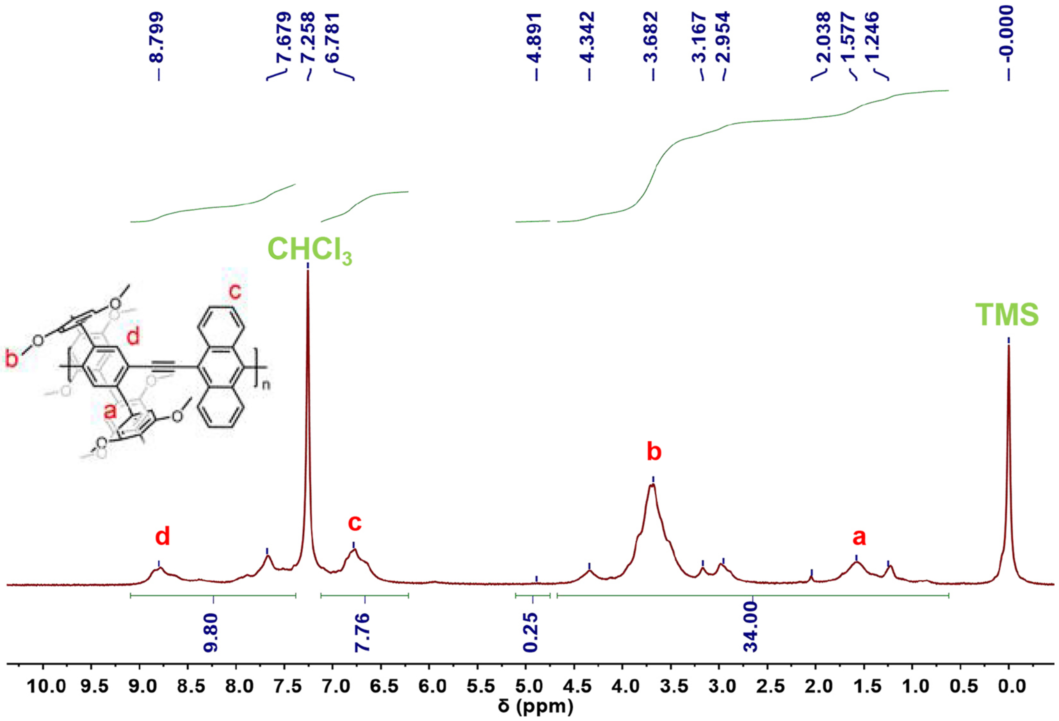

Figure S8. ^{1}H NMR spectrum (400 MHz, $CDCl_3$, 298 K) of p(P[5]$(OTf)_2$-*co*-9,10-dea); Yield calculation: (polymer characteristic peak area/substrate characteristic peak area)×100%. Mn calculated by ^{1}H NMR: Mn=$(H_d/10)/H_{\text{terminal alkyne}}\times M_{\text{repeating units}}$.

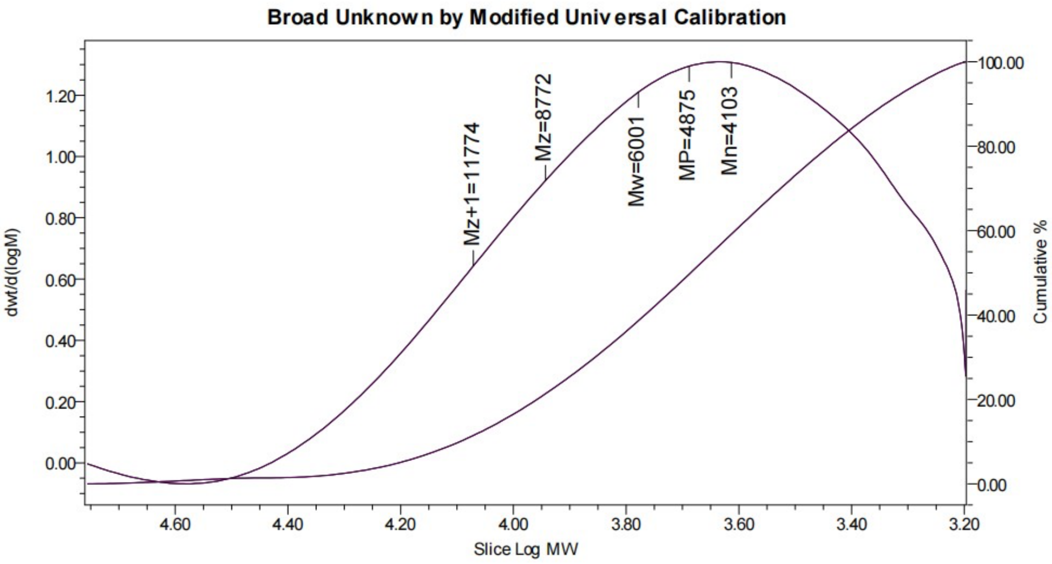

Figure S9. GPC trace of p(P[5]$(OTf)_2$-*co*-9,10-dea).

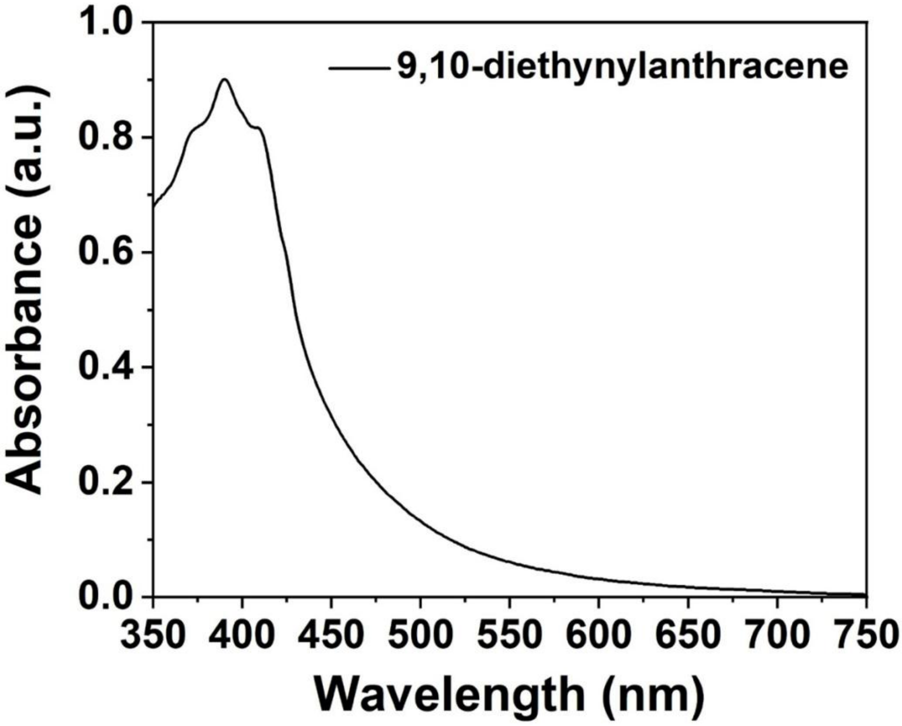

Figure S10. UV-vis absorption spectrum of 9,10-diethynylanthracene in THF. [Pillar[5]arene unit] = 10^{-3} M; (slit widths: ex. 5 nm, em. 3 nm; 25 ℃).

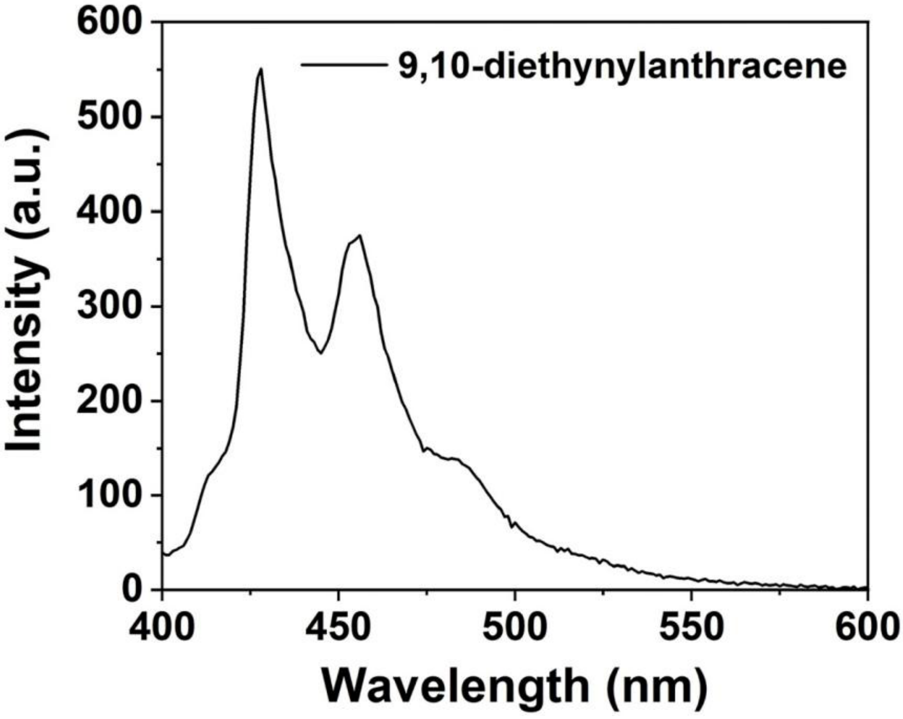

Figure S11. Fluorescent emission spectrum of 9,10-diethynylanthracene in THF. [Pillar[5]arene unit] = 10^{-4} M; (λ_{ex} = 390 nm; λ_{em} = 428, 455 and 480 nm; slit widths: ex. 5 nm, em. 3 nm; 25 ℃).

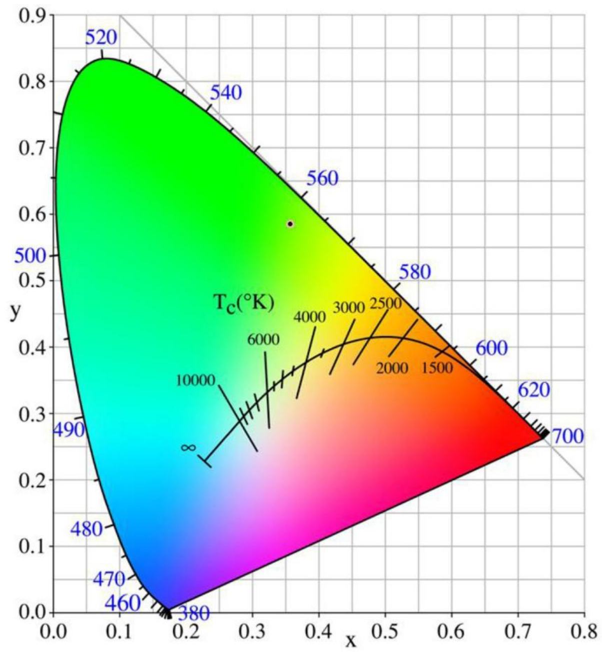

Figure S12. CIE chromaticity diagram for p(P[5](OTf)$_2$-*co*-9,10-dea).

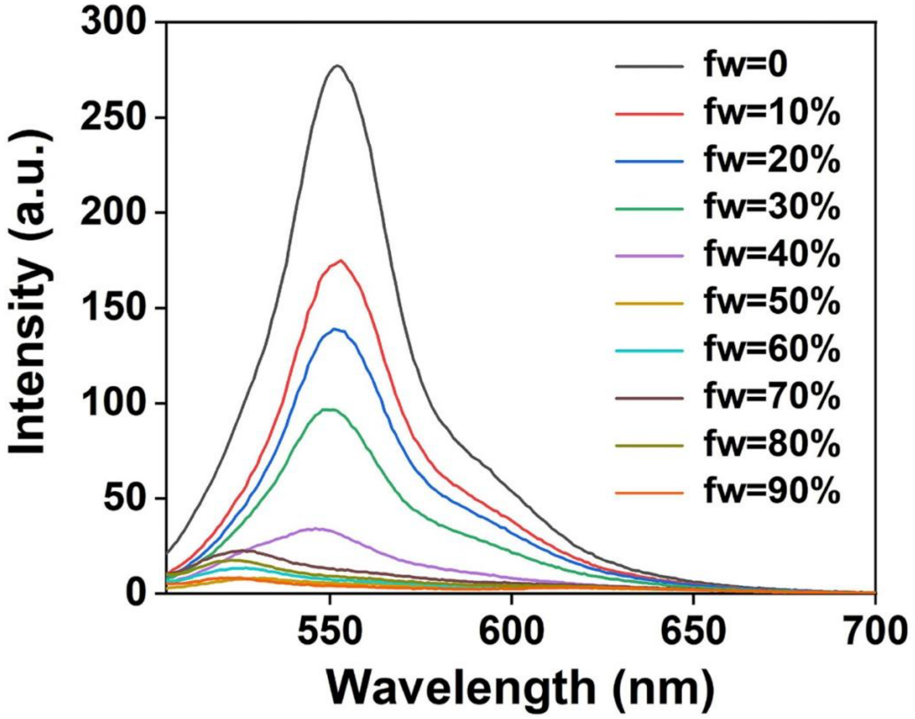

Figure S13. The fluorescence spectra of p(P[5](OTf)$_2$-*co*-9,10-dea) with different formula weight (fw) of water in THF. [Pillar[5]arene unit] = 10^{-5} M; (λ_{ex} = 480 nm; λ_{em} = 552 nm; slit widths: ex. 5 nm, em. 3 nm; 25 °C).

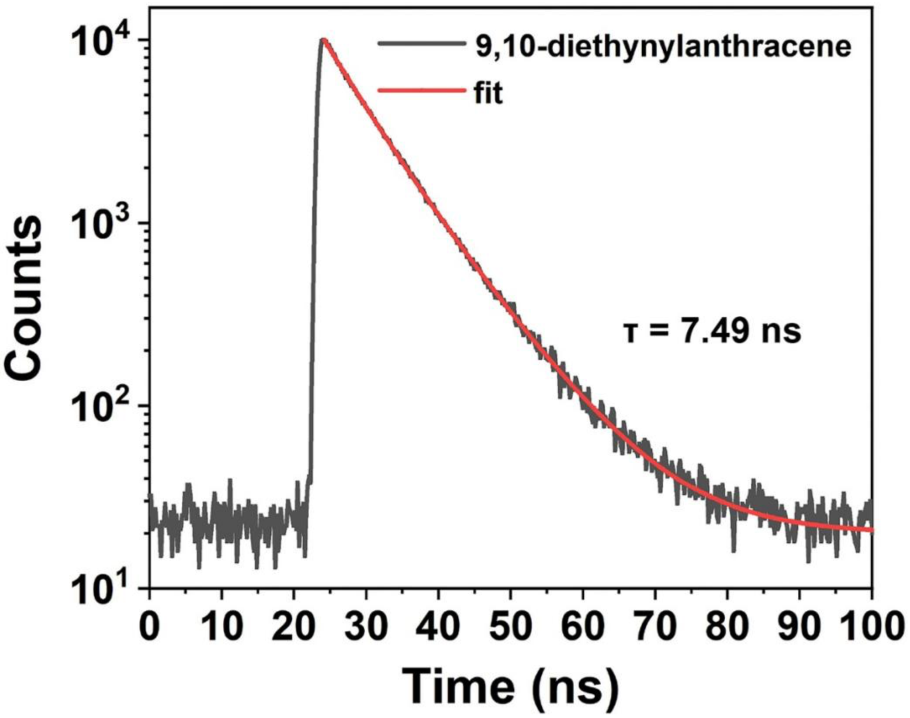

Figure S14. Fluorescence decay profile of 9,10-diethynylanthracene; (solution: THF; λ_{ex} = 400 nm; [pillar[5]arene unit] = 10^{-5} M, 25 °C).

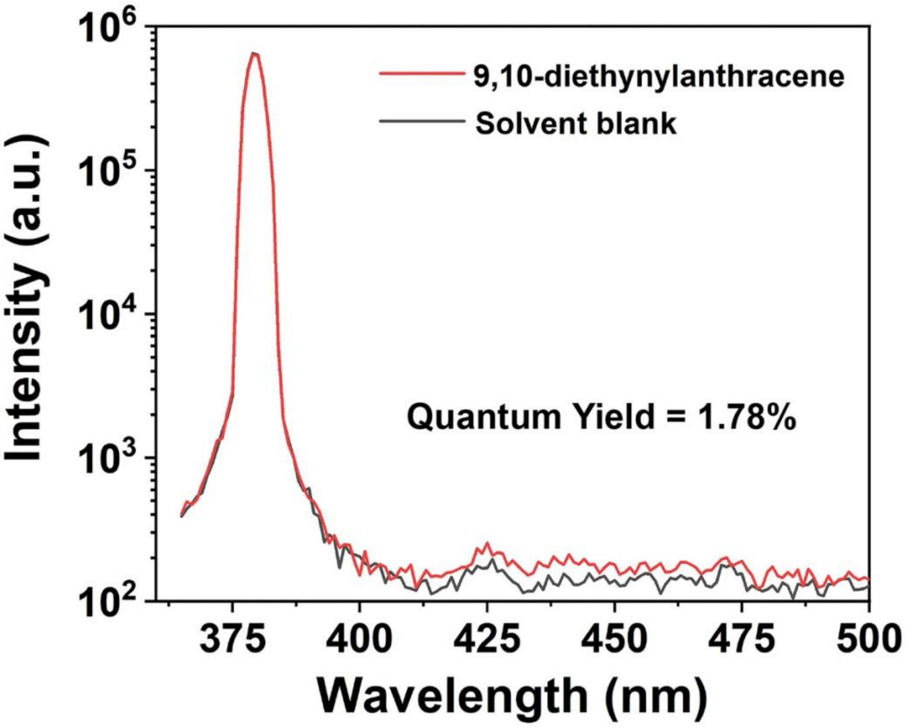

Figure S15. Fluorescence quantum yield of 9,10-diethynylanthracene in THF solvent; (λ_{ex} = 400 nm, [pillar[5]arene unit] = 10^{-5} M, 25 °C).

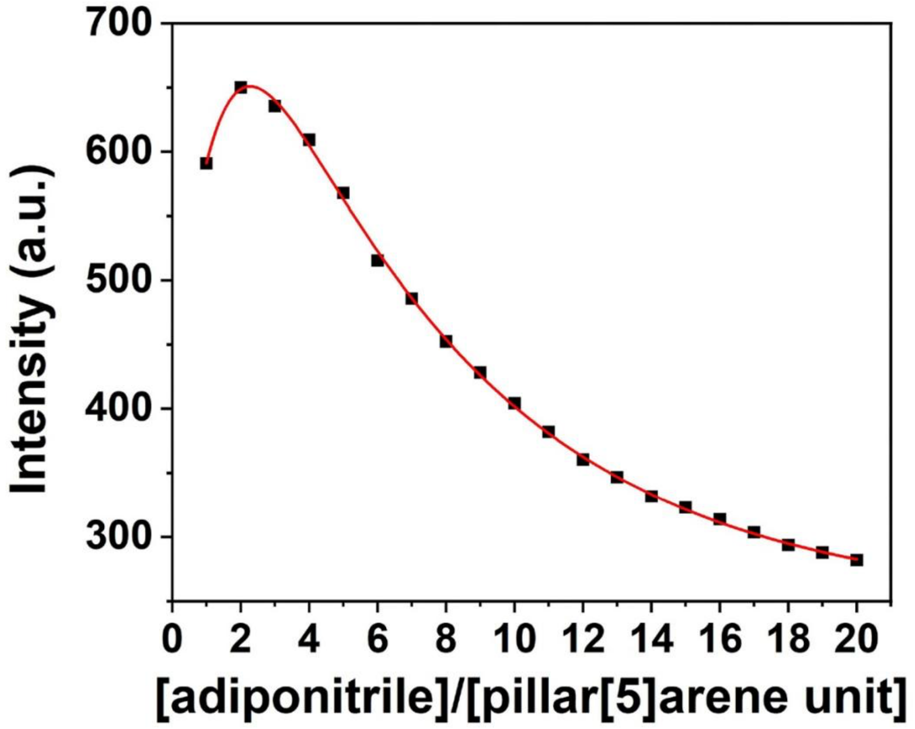

Figure S16. Fluorescence fitting curves of p(P[5](OTf)2-*co*-9.10-dea) conjugated polymers in THF with different ratios of adiponitrile; (λ_{ex} = 480 nm; λ_{em} = 552 nm; slit widths: ex. 5 nm, em. 3 nm; 25 °C).

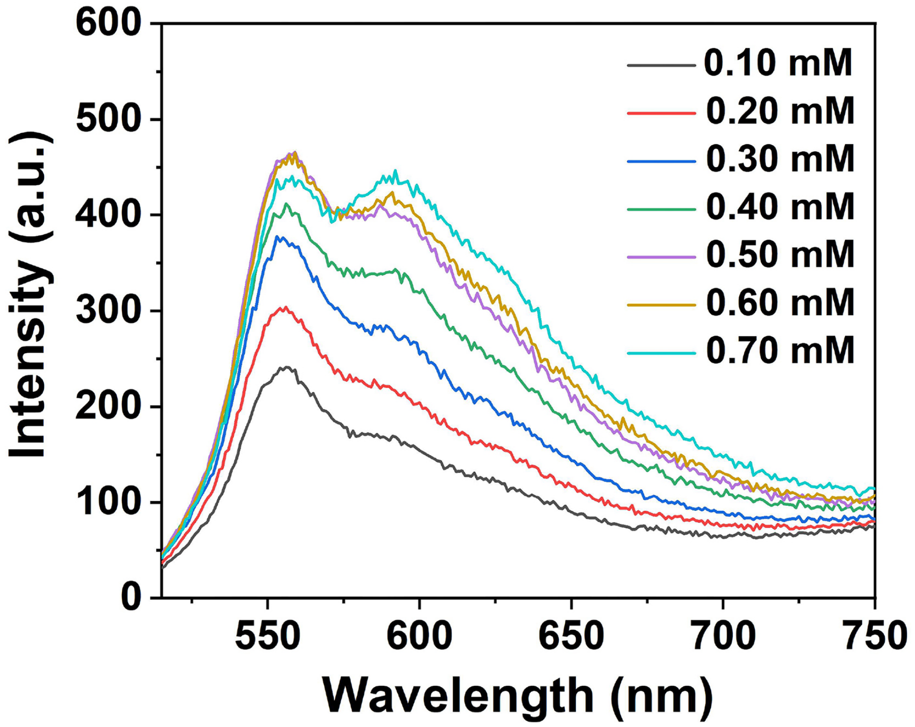

Figure S17. Fluorescence spectra of p(P[5](OTf)2-*co*-9,10-dea) CP in THF with increasing concentration from 0.1 mM to 0.7 mM. (slit widths: ex. 3 nm, em. 1.5 nm; 25 °C).

References

S1. Li, Q.; Zhu, H.T.Z.; Huang, F.H. Alkyl chain length-selective vapor-induced fluorochromism of pillar[5]arene-based nonporous adaptive crystals. *J. Am. Chem. Soc.* **2019**, *141*, 13290-13294.

S2. Ju, J.U.; Chung, D.S.; Kim, S.O.; Jung, S.O.; Park, C.E.; Kim, Y.-H.; Kwon, S.-K. Synthesis and characterization of a new ethynyl-linked alternating anthracene/fluorene copolymer for organic thin film transistor. *J. Polym. Sci. Part A: Polym. Chem*. **2009**, *47*, 1609-1616.
